# An Atypical Presentation of a Polyarticular Gout Flare: Case Report

**DOI:** 10.7759/cureus.46967

**Published:** 2023-10-13

**Authors:** Larua Londono, Michael Makutonin, Anthony Dure, Justin Canakis, Luis W Dominguez

**Affiliations:** 1 Internal Medicine, George Washington University School of Medicine and Health Sciences, Washington, USA; 2 Internal Medicine, George Washington University Hospital, Washington, USA; 3 Internal Medicine/Primary Care, George Washington University Hospital, Washington, USA

**Keywords:** sepsis-like syndrome, polyarticular, gout, diagnostic challenge, gout flare

## Abstract

A 54-year-old man with a history of hypertension, atrial fibrillation, chronic kidney disease, nonischemic cardiomyopathy, osteoarthritis, and gout presented to the emergency department (ED) with dysuria, painful scrotal swelling, severe bilateral flank pain, back pain, atraumatic right arm (elbow and distally) pain and swelling, and bilateral knee pain. His physical exam was notable for fever, tachycardia, bilateral costovertebral angle (CVA) tenderness, exquisite pain, erythema, and swelling of bilateral knees and the right arm (elbow and distally). He met Systemic Inflammatory Response Syndrome (SIRS) criteria, was placed on Ceftriaxone for presumed septic pyelonephritis, and was admitted to the medicine team. With initially unremarkable imaging studies, the differential diagnosis was broadened, and subsequent infectious workups yielded grossly normal results. At the end of hospital day one, the patient remained febrile and without symptomatic improvement. Rheumatology was consulted and empirically treated; the patient with a dose of Anakinra due to concerns about a polyarticular flare of crystalline arthropathy. Subsequent arthrocentesis confirmed a final diagnosis of a polyarticular gout flare. This case highlights the diagnostic challenges a polyarticular gout flare poses and the importance of early involvement of specialists for prompt recognition, treatment, and avoidance of unnecessary interventions.

## Introduction

Gout is the most common form of inflammatory arthritis in the world, with a global prevalence of 3% and 6% [1]. It is important to be able to diagnose and treat early to prevent the pain burden, disability, and nephropathy possible with untreated disease [2]. During an acute gout flare, it is common for one joint to be affected, but polyarticular gout flares (defined as pain affecting multiple joints due to monosodium urate crystal deposition) can also occur [3]. The gold standard for diagnosing gout is a synovial fluid analysis of the affected joints [4], which is often missed due to atypical presentation and significant pain.

This case report aims to showcase a unique presentation of polyarticular gout with a systemic inflammatory state that poses diagnostic challenges and highlights the importance of early collaboration with specialists. Few other case reports have been published demonstrating the wide array of possible presentations of a polyarticular gout flare and, thus, the diagnostic challenges associated [5-9]. This report was previously presented at the New England Journal of Medicine Research Symposium at the Latino Medical Student Association-North East 50 Conference, and at the George Washington University Medical Student Research Day.

## Case presentation

A 54-year-old man with a history of hypertension, atrial fibrillation, chronic kidney disease (CKD) stage 3, nonischemic cardiomyopathy, osteoarthritis, and gout presented with dysuria, painful scrotal swelling, severe bilateral flank pain, back pain, atraumatic right arm (elbow and distally) pain and swelling, and bilateral knee pain. Home medications for his chronic conditions included amiodarone, apixaban, bumetanide, carvedilol, empagliflozin, and hydralazine. He stated he was not currently taking daily urate-lowering medications for his gout. Notably, this patient had a history of a polyarticular gout flare three months before the current presentation, requiring hospitalization, and was discharged on fexubostat and colchicine. For unknown reasons, he had not received these medications and was not taking them. He reported a historical allergy (rash) to allopurinol.

His chief complaint was bilateral flank pain, stating he was unable to walk due to severe "kidney pain", as well as having diffuse pain in his right upper extremity and bilateral knees. He endorsed the worsening of his chronic back pain. He also mentioned a sore throat for a few days. He denied any alcohol intake, new sexual partners, or recent injury to the affected joints. On arrival, he was febrile (39.3C), tachycardic (111 bpm), and in significant distress. The physical exam was notable for bilateral CVA tenderness. Bilateral knees and the right upper extremity from elbow to fingertips were erythematous, edematous, warm, and exquisitely tender, with range of motion significantly limited due to pain. There were no overlying skin lesions, rashes, or obvious deformities of the affected joints. His scrotum was tender and enlarged, with bilateral testicles palpated.

Laboratory analysis, as shown in Table 1, demonstrated a remarkably elevated erythrocyte sedimentation rate (ESR) and C-reactive protein (CRP), a white blood cell (WBC) count of 24,000, and renal markers of creatinine and glomerular filtration rate consistent with the patient's baseline. Given his tachycardia and fever, he met SIRS criteria for sepsis. Abdominal computed tomography (CT) scans and X-rays of all involved joints were normal, and urine was sent for urine analysis. The patient was treated with ceftriaxone for presumed septic pyelonephritis and admitted to medicine.

**Table 1:** Initial laboratory workup in the ED ESR: Erythrocyte sedimentation rate, CRP: C-reactive protein, GFR: Glomerular filtration rate

Lab Investigation	Lab Value	Reference Range
White blood cell (WBC) count	23.87 x10e3/mcL	4.5 to 11.0 x10e3/mcL
Erythrocyte sedimentation rate (ESR)	108 mm/hr	< 20 mm/hr
C-reactive protein (CRP)	265.4 mg/L	< 10 mg/L
Serum creatinine	2.5 mg/dL	0.74 to 1.35 mg/dL
Glomerular filtration rate (GFR)	28 mL/min	> 60 mL/min

After 24 hours, the patient remained febrile without symptom improvement; he had persistent leukocytosis and no clear source of infection. Antibiotics were broadened to include vancomycin and zosyn. The differential at this time included right upper extremity deep vein thrombosis (DVT), disseminated gonococcal infection, and septic arthritis with seeding from spinal foci due to back pain. The workup by both the Emergency Medicine and Internal Medicine teams, including urinalysis, blood and urine cultures, abdominal/spinal CT, scrotal and right upper extremity ultrasound, a sexually transmitted infection panel, and X-rays of involved joints, was unremarkable. Rheumatology was consulted for evaluation and possible arthrocentesis at the end of hospital day one. 

Rheumatology differential diagnosis included: septic arthritis due to high fevers, leukocytosis, and positive clinical signs; osteomyelitis due to acute worsening of back pain, leukocytosis, fevers, and involvement of multiple joints; pseudogout due to a history of osteoarthritis and positive clinical signs; and gout due to risk factors such as heart failure, chronic kidney disease, obesity, and diuretics. A serum uric acid level was elevated at 9.4 mg/dL (reference range 3.5-7.2 mg/dL). The plan was to continue current antibiotics and await final blood cultures, empirically administer Anakinra 100 mg subcutaneously for a possible crystalline arthropathy flare and reassess the following day. 

On hospital day two, the patient's pain had improved, and he tolerated movement of his upper extremities. The complete blood cultures came back negative. The patient had worsening renal function, and anakinra dosing was reduced to every other day for nephroprotection. The patient continued to refuse knee arthrocentesis due to pain despite extensive counseling. The pain regimen was subsequently broadened to include lidocaine patches and, as needed, short-acting opioids. 

On the third day of admission, bilateral knee arthrocentesis revealed cloudy fluid with uric acid crystals and an elevated WBC count, confirming a final diagnosis of polyarticular gout flare (Table 2). Anakinra dosing was continued throughout the patient’s hospital stay. It was hypothesized that this patient's current polyarticular gout flare had a multifactorial etiology, including his lack of urate-lowering prophylactic therapy and multiple risk factors (obesity, diuretics, heart failure, and CKD). 

**Table 2:** Synovial fluid analysis of bilateral knee joint aspirate WBC: White blood cell count, RBC: Red blood cell count

Synovial Fluid Analysis	Value	Reference Range
Appearance	Cloudy	Transparent
White blood cell count (WBC)	4347 mm^3^	< 200 x 106/L
Red blood cell count (RBC)	2000 mm^3^	< 2000 mm^3^
Crystals	Uric acid crystals	None

Upon discharge to an acute rehabilitation center, rheumatology recommended taking febuxostat 20 mg daily with a goal of increasing to 40 mg daily if kidney function allowed, along with colchicine 0.5 mg every other day. Febuxostat was chosen due to the patient's drug allergy to allopurinol. A follow-up appointment with rheumatology was scheduled for the following month. 

## Discussion

Gout is the most common form of inflammatory arthritis, with a classic presentation most practitioners are familiar with [1]. Contrary to the classic presentation of monoarticular gout flares, polyarticular gout flares can trigger a systemic inflammatory state. Depending on which joints are involved greatly changes the initial clinical presentation and diagnostic acumen. Despite this severe and potentially misleading presentation, comparatively few case studies have elucidated the variability in the presentation of polyarticular gout and its diagnostic challenges [6,7]. 

Patients suffering from a polyarticular gout flare may meet SIRS criteria for sepsis, which may lead to anchoring on a septic source and workup. In the case presented, despite an unremarkable infectious workup, the patient was placed on broad-spectrum antibiotics and continued on the regimen for several days without symptomatic improvement. A rheumatology consult eventually resulted in empiric Anakinra administration and an improved symptom burden for the patient on hospital day two.

While the patient had numerous risk factors for severe gout and a documented history of gout and a polyarticular gout flare, a polyarticular gout flare was not on the initial differential due to his multisystem and polyarticular involvement. Appropriately, diagnostic preference was first given to rule out serious infectious causes of the patient’s presentation. However, a polyarticular gout flare should not be treated as a diagnosis of exclusion for patients presenting in a pain crisis with an unremarkable initial infectious workup. Instead, the authors of this case report recommend considering a polyarticular gout flare alongside other, more life-threatening diagnoses. As with the patient described here, if a polyarticular gout flare is the true diagnosis, the patient will not improve on treatments targeting an infectious etiology and may experience iatrogenic harm due to physiologic responses to antibiotics and hyperuricemia. The authors recommend early laboratory analysis of synovial fluid aspirated from affected joints and early consultation with rheumatology. Not only does this ensure timely diagnosis and appropriate treatment, but it can also prevent unnecessary interventions like prolonged intravenous antibiotics and procedures [8-9], which increase the risk for further complications and lead to an increased use of hospital resources. 

Diagnosing polyarticular gout flares remains challenging despite increasing awareness through case report publications. The variability in affected joints, acuity of presentation, lack of acutely inciting factors, and confounding findings can lead providers away from the correct diagnosis. While a recent study has found an association between neutrophil activation markers such as peroxidase and calprotectin and polyarticular gout flares [10], there is no statistically validated laboratory value to tip physicians towards this diagnosis outside of birefringent crystals found on joint aspirate. More research is necessary to be able to better identify and streamline the diagnostic path for these patients. 

## Conclusions

Polyarticular gout flares pose significant diagnostic challenges for providers, and the delay in diagnosis subjects these patients to prolonged pain, unnecessary procedures, medications, and complications. This case highlights the importance of maintaining a broad, flexible differential for patients without a straightforward diagnostic path and the early involvement of specialists to facilitate a timely diagnosis and correct treatment.
